# Controlling malignant pericardial effusion by intrapericardial carboplatin administration in patients with primaryon-small-cell lung cancer

**DOI:** 10.1054/bjoc.2000.1397

**Published:** 2000-09-04

**Authors:** T Moriya, Y Takiguchi, H Tabeta, R Watanabe, H Kimura, K Nagao, T Kuriyama

**Affiliations:** Department of Chest Medicine, Chiba University School of Medicine, Chiba, Japan; Department of Chest Medicine, Funabashi Medical Center, Funabashi, Japan; Health Sciences Center, Chiba University, Chiba, Japan

**Keywords:** cardiac tamponade, pericardial effusion, malignant pericarditis, lung cancer, carboplatin, pharmacokinetics

## Abstract

Malignant pericarditis, when associated with massive pericardial effusion, presents a critical condition in lung cancer patients. Because this condition often arises in terminally ill patients, intensive therapy such as multi-drug combination chemotherapy is rarely appropriate. This study evaluated the clinical relevance of intrapericardial administration of carboplatin for controlling malignant pericardial effusions associated with non-small-cell lung carcinoma (NSCLC). The method used for 10 eligible patients consisted of draining the pericardial effusion and infusing 300 mg/body of carboplatin in 50 ml of saline through an in-place catheter into the pericardial space and clamping the catheter for 40 min. Nine of the 10 patients showed satisfactory results, and 8 experienced complete regression of the effusion. No major or minor adverse effects were observed. Pharmacokinetics analysis revealed that the concentration of free platinum in the pericardial fluid was very high while that of total platinum in the circulating plasma was very low, assuring the usefulness of the intrapericardial instillation of carboplatin in terminally ill patients for controlling malignant pericardial effusion when the systemic delivery of cytotoxic agents is inappropriate. © 2000 Cancer Research Campaign


*British Journal of Cancer* (2000) **83**(7), 858–862
© 2000 Cancer Research Campaign
doi: 10.1054/ bjoc.2000.1397, available online at http://www.idealibrary.com on IDEAL®

### Controlling malignant pericardial effusion by intrapericardial carboplatin administration in patients with primary non-small-cell lung cancer

**T Moriya, Y Takiguchi, H Tabeta*, R Watanabe, H Kimura, K Nagao** and T Kuriyama**

Department of Chest Medicine, Chiba University School of Medicine Chiba, Japan; *Department of Chest Medicine, Funabashi Medical Center Funabashi, Japan; **Health Sciences Center, Chiba University Chiba, Japan

**Summary** Malignant pericarditis, when associated with massive pericardial effusion, presents a critical condition in lung cancer patients. Because this condition often arises in terminally ill patients, intensive therapy such as multi-drug combination chemotherapy is rarely appropriate. This study evaluated the clinical relevance of intrapericardial administration of carboplatin for controlling malignant pericardial effusions associated with non-small-cell lung carcinoma (NSCLC). The method used for 10 eligible patients consisted of draining the pericardial effusion and infusing 300 mg/body of carboplatin in 50 ml of saline through an in-place catheter into the pericardial space and clamping the catheter for 40 min. Nine of the 10 patients showed satisfactory results, and 8 experienced complete regression of the effusion. No major or minor adverse effects were observed. Pharmacokinetics analysis revealed that the concentration of free platinum in the pericardial fluid was very high while that of total platinum in the circulating plasma was very low, assuring the usefulness of the intrapericardial instillation of carboplatin in terminally ill patients for controlling malignant pericardial effusion when the systemic delivery of cytotoxic agents is inappropriate. © 2000 Cancer Research Campaign

**Keywords:** cardiac tamponade; pericardial effusion; malignant pericarditis; lung cancer; carboplatin; pharmacokinetics

Occasionally, both advanced small-cell lung cancers (SCLC) and non-small-cell lung cancers (NSCLC) can cause carcinomatous pericarditis. Malignant pericarditis is critically and inevitably associated with pericardial effusion which, in turn, incites cardiac tamponade (Press and Livingston, 1987). This is a life-threatening situation especially in terminal cases because malignant cardiac tamponade invariably results in acute cardiac failure. Therefore, by controlling pericardial effusion, the quality of life of these terminally ill patients may be improved.

Malignant pleuritis in patients having SCLC is effectively controlled by systemic, multi-drug chemotherapy (Livingston et al, 1982; Herrstedt et al, 1992). Accordingly, the most prudent option for controlling primary and metastatic lesions in these cases, or even for controlling malignant pericarditis, is a combination of systemic chemotherapy and effusion extraction to relieve cardiac tamponade. For patients with NSCLC, however, a systemic chemotherapeutic regimen generally has a relatively brief and low response rate of 20–50% (Woods et al, 1990; Souquet et al, 1993), making regional treatment of malignant pericarditis much more meaningful. In addition, since this condition arises in terminally ill patients having NSCLC (Press and Livingston, 1987), intensive therapy such as combination chemotherapy often cannot be considered. The most commonly applied treatment for malignant pericarditis in NSCLC patients consists of simple drainage of the pericardial effusion (Shinkai et al, 1982; Roth et al, 1989), although it is not without controversy (Okamoto et al, 1993). The topical instillation of some kinds of chemotherapeutic or sclerosing agents has at times been performed in an attempt to achieve a better outcome (Smith et al, 1974; Shepherd et al, 1987; Celermajor et al, 1991; Vaitkus et al, 1994; Maher et al, 1996). Especially, Maher et al reported results from a large-scale study of 93 patients with malignant pericarditis and cardiac tamponade caused by a variety of malignant diseases including 45 lung cancers. They employed the intrapericardial administration of tetracycline hydrochloride or doxycyclin hydrochloride and suggested that pericardial sclerosis provided similar survival with lower mortality and recurrence rates when compared to the outcomes of drainage by video-assisted thoracoscopic surgery or subxiphoid pericardial window creation with tube drainage. Unfortunately, the fact that there has been no systematic prospective investigation has prevented the confirmation of this advantage, and any such alternative method must first be evaluated for both its efficacy and safety. Only then can conclusions be drawn from phase III studies in which the two methods are directly compared. For designing phase III studies, alternative methods including the topical instillation of cytotoxic agents need to be investigated in either a pilot study or a phase II study.

This report describes the usefulness and pharmacokinetics data of topically injected carboplatin for patients with NSCLC and malignant cardiac tamponade.

### PATIENTS AND METHODS

### Eligibility criteria for selecting patients

Ten patients, having met all of the following conditions, were enrolled in the study: 1. histologically and/or cytologically proven NSCLC, 2. massive pericardial fluid proven to contain malignant

*Received 7 December 1999*
*Revised 26 June 2000*
*Accepted 4 July 2000*

*Correspondence to:* Y Takiguchi cells, 3. cardiac tamponade diagnosis made by means of suggestive symptoms and physical signs, electrocardiograms, chest X-rays, echo cardiograph and CT scan images, 4. age from 16 to 80 years, 5. performance status (PS) from 1 to 4, 6. normal bone marrow function with Hb ≥ 9 g/dl, WBC ≥ 3000/μl, Plt ≥ 100 000/μl, tolerable liver function showing serum GOT and GPT within two-fold the normal value, and normal renal function showing serum creatinine not exceeding 1.5 mg/dl, 7. expected survival period of more than four weeks if cardiac tamponade could be controlled, 8. no chemotherapy, radiotherapy or surgery at least one month prior to the treatment, and 9. written informed consent. This study fully complies with institutional regulations.

### Treatment method

After laying the patient down in a semi-Furler position and applying local anaesthesia, a 5 French or 16 Gauge catheter was percutaneously inserted into the pericardial cavity under echocardiographic guidance. The effusion was completely drained through the catheter, and the cavity was then washed several times with heparinized saline. After infusing 300 mg of carboplatin and 100 mg of lidocaine dissolved in 50 ml of normal saline via the catheter into the pericardial cavity, the catheter was then occluded for 40 minutes. During this period the patients could opt for supine, right-sided, left-sided and prone positions for 10 minutes each. Upon reopening the catheter, any remaining effusion was manually drained once a day until it reached less than 30 ml/day, at which point the catheter was removed. In cases where the catheter could not be removed within five days following the treatment, carboplation was administered one more time.

### Definition of effectiveness

Since terminally ill patients with advanced NSCLC are survival-limited, the following criteria were used to evaluate the advantages of this treatment.

### *Major response*

No continuous drainage requirement, no pericardial effusion recurrence and no signs of cardiac tamponade requiring further treatment for at least 4 weeks and/or until expiration.

### *Moderate response*

No continuous drainage requirement, no significant pericardial effusion recurrence and no signs of cardiac tamponade requiring further treatment for at least 4 weeks, but drainage reinstallation being required beyond this period.

### *No response*

Continuous drainage requirement or drainage reinstallation during the first 4 weeks.

### Pharmacokinetics study

Circulating venous blood and pericardial fluid samples were collected in heparinized tubes at 0, 15, 30 minutes, and 1, 2, 4, 8, 16 and 32 hours after reopening the drainage catheter. All samples were directly centrifuged at 3000 rpm for 20 minutes to isolate the plasma. A 2 ml portion of each plasma gradient was then placed on a Centrifree MPS-3 conical filter (Amicon Corp., Lexington, Mass., USA) and centrifuged again at 3000 rpm for 20 minutes to eliminate existing protein and protein-bound platinum. A preliminary study revealed that this filtered plasma was transparent and contained less than 2% plasma protein (data not shown). The filtered and unfiltered samples were stored at –70°C until measurement. All samples were measured for platinum concentrations by Flameless Atomic Absorption Spectroscopy using the same instrumentation and method as reported earlier (Pera and Harde, 1977). By this analysis, the lowest detectable platinum concentration was 50 ng/ml.

Then, the measured platinum concentrations were fitted to a two-compartment open model using a nonlinear regression program based on a simplex algorithm. Justification for choosing this particular model has been previously established (Patton et al, 1978).

### RESULTS

### Effectiveness for controlling malignant effusion

The characteristics of the 10 patients are summarized in Table 1. All patients had adenocarcinoma and the median age of the 6 men and 4 women was 54 years (range 44 to 72). Although nine patients unquestionably had clinical stage IV disease at the time of cardiac tamponade diagnosis, the remaining patient (case 2 in Table 1), diagnosed with clinical stage IIIB or IV, refused to be completely evaluated for clinical stage. PS of the 10 patients is also shown in Table 1. Each patient deteriorated to a PS of 4 because of cardiac tamponade.

**Table 1** Patient characteristics and results

| Patient no. | Age[a] | Sex | Cell type | Clinical stage | PS | No. of administrations of carboplatin | Duration of drainage (days) | Response | Survival[c] (days) |
|---|---|---|---|---|---|---|---|---|---|
| 1 | 44 | Male | Ad[b] | T4N3M1 | 4 | 1 | 6 | Major | 29 |
| 2 | 49 | Male | Ad | T4N3Mx | 4 | 2 | 13 | Major | 53 |
| 3 | 52 | Male | Ad | T4N3M1 | 4 | 1 | 6 | Major | 79 |
| 4 | 51 | Female | Ad | T4N3M1 | 4 | 1 | 4 | Major | 59 |
| 5 | 56 | Male | Ad | T4N3M1 | 4 | 1 | 5 | Major | 38 |
| 6 | 58 | Male | Ad | T4N3M1 | 4 | 2 | 11 | Major | 176 |
| 7 | 63 | Male | Ad | T4N3M1 | 4 | 2 | 16 | Major | 100 |
| 8 | 57 | Female | Ad | T4N3M1 | 4 | 2 | 8 | Major | 112 |
| 9 | 48 | Female | Ad | T4N3M1 | 4 | 2 | 14 | Moderate | 158 |
| 10 | 72 | Female | Ad | T4N3M1 | 4 | 2 | 31 | No response | 32 |

[a] Median age, 54 years; [b] adenocarcinoma of lung; [c] median survival time from the start of CBDCA intrapericardial administration, 69 days.

The result of the intrapericardial administration of carboplatin and successive draining of pericardial effusions was favourable. Among the 10 cases, there were 8 major responders, one moderate responder and one non-responder (Table 1). As for the 4 major responders (cases 1, 3, 4 and 5) who required only one injection of carboplatin, the drainage tubes were removed within 6 days after treatment without the recurrence of pericardial effusion. The other four major responders (cases 2, 6, 7 and 8) required a second injection of carboplatin to achieve a major response. Case 1 expired at 29 days after treatment because of tumour haemoptysis. Case 9 experienced a pericardial effusion recurrence 89 days after the first carboplatin injection and, being judged as a moderate responder, was subsequently treated again. This re-injection of carboplatin was effective for another 67 days until her death. Case 10 required continuous pericardial drainage of more than 30 ml for 31 days after treatment or until her death, and was consequently judged as a non-responder. The overall median survival time for these ten patients was 69 days (ranging from 29 to 176 days).

### Adverse effects

No significant adverse effects such as fever, infection, retrosternal pain, new arrhythmia, or deterioration of laboratory data were observed during this study. Although the effused pericardial fluid was moderately viscous in most patients, the drainage tube only rarely became blocked, and then the obstruction was easily flushed out with heparinized saline.

All 10 patients died because of progressing cancer, but they were without any clinical symptoms related to cardiac tamponade at the time of their passing. Most importantly, no evidence of pericardial constriction was ever observed.

### Carboplatin pharmacokinetics in pericardial fluid and circulating blood

Seven cases (cases 1, 2, 3, 5, 7, 9 and 10) gave carboplatin pharmacokinetics data in pericardial fluid and four cases (cases 3, 5, 7 and 9) gave the data in circulating blood. The pharmacokinetics parameters of platinum are given in Table 2 and the mean decay curves for platinum in pericardial fluid and circulating plasma are shown in Figures 1A and 1B, respectively.

Peak concentrations of platinum in pericardial fluid were reached immediately following intrapericardial administration, after which they declined (Fig. 1A). The area under the concentration–time curve (AUC) of total platinum in pericardial fluid was 462.8 ± 253.1 μg × h/ml and that of free platinum 385.1 ± 221.7 μg × h/ml (Fig. 1A and Table 2). Concentrations of total platinum in pericardial fluid declined in a bi-exponential fashion with α and β half-lives of 0.52 ± 0.06 and 9.93 ± 7.07 hours, respectively. Concentrations of ultrafiltrable platinum, or free platinum, also declined in a bi-exponential fashion with α and β half-lives of 0.46 ± 0.09 and 5.06 ± 2.23 hours, respectively. The percentage of free platinum in pericardial fluid was more than 70% one hour after reopening the catheter, then declining to 50% at 8 hours.

The total platinum and free platinum concentrations in plasma declined in a bi-exponential fashion. Platinum concentrations in circulating blood showed a peak at 15 min after beginning drainage. The maximum concentration ($C_{max}$) of serum total platinum was 5.8 ± 4.8 μg/ml, or 0.8% of that in pericardial fluid. AUC of total platinum in plasma fluid was 33.3 ± 14.0 μg × h/ml and that of free platinum was 11.3 ± 4.1 μg × h/ml (Fig. 1B and Table 2).

**Table 2** Pharmacokinetic parameters of platinum in pericardial fluid and plasma

| | | $C_{max}$ (μg/ml) | AUC (μg h/ml) | t1/2$^{\alpha}$ (h) | t1/2$^{\beta}$ (h) |
|---|---|---|---|---|---|
| Pericardial fluid | total platinum | 715.8 ± 241.9 | 462.8 ± 253.1 | 0.52 ± 0.06 | 9.93 ± 7.07 |
| | free platinum | 643.0 ± 241.6 | 385.1 ± 221.7 | 0.46 ± 0.09 | 5.06 ± 2.23 |
| Plasma | total platinum | 5.8 ± 4.8 | 33.3 ± 14.0 | 1.92 ± 0.60 | 20.89 ± 9.97 |
| | free platinum | 3.8 ± 1.5 | 11.3 ± 4.1 | 1.86 ± 0.70 | 2.56 ± 0.20 |

$C_{max}$: Maximum concentration; AUC: area under the concentration-time curve; T1/2$^{\alpha}$: α-phase half-life; T1/2$^{\beta}$: β-phase half-life.

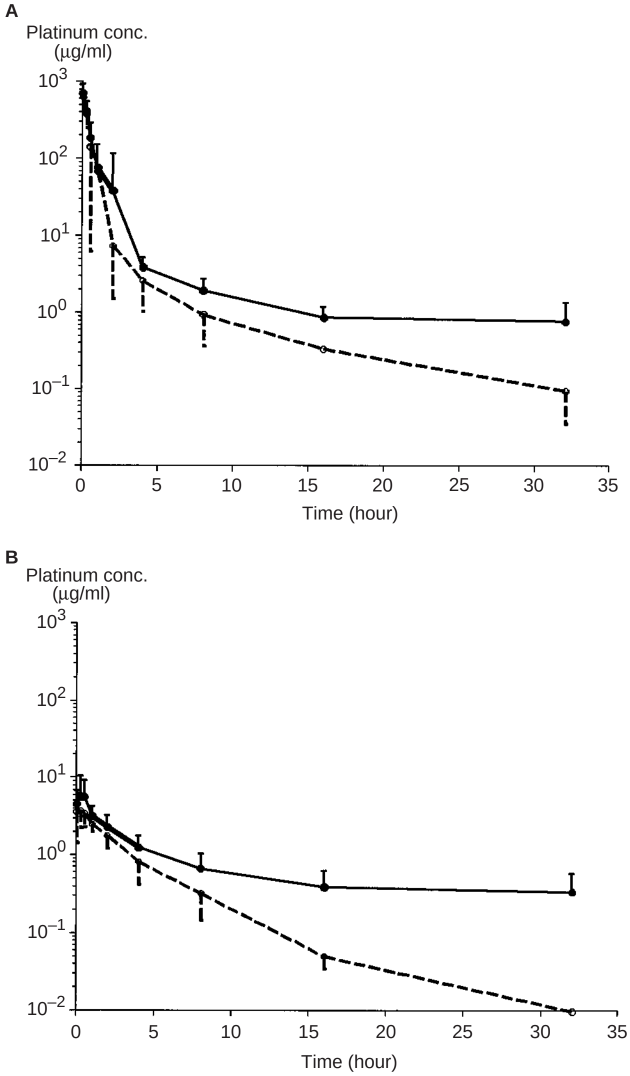

**Figure 1** Platinum concentration in intrapericardial space (**A**) and in circulating plasma (**B**), in patients treated with intrapericardial administration of carboplatin. Fluid samples were collected at 1, 15, 30 minutes and 1, 2, 4, 8, 16, 32 hours after reopening the occluded drainage catheter. The data consists of 7 patients in **A** and 4 patients in **B**. Each point represents the mean and each bar represents the standard deviation of the total platinum (solid line) and free platinum (dotted line)

 *© 2000 Cancer Research Campaign*

**Table 3** Summary of published studies on typical administration of sclerosing or cytotoxic agents for treating malignant pericardial effusion

| Agent | Investigator[a] | Year | Efficiency[b,c] | Adverse effects |
|---|---|---|---|---|
| Tetracycline hydrochloride | Davis et al | 1984 | 30/33 (91%) | None |
| | Shepherd et al | 1987 | 43/58 (74%) | Pain, fever, arrhythmia |
| | Celermajor et al | 1991 | 19/26 (73%) | Pain |
| Doxycycline hyclate | Kitamura et al | 1981 | 7/7 (100%) | Pain |
| Doxycycline hydrochloride | Liu et al | 1996 | 6/9 (67%) | Pain, fever, arrhythmia |
| Tetracycline hydrochloride or doxycycline hydrochloride | Maher et al | 1996 | 68/85 (79%) | Pain, fever, arrhythmia |
| Bleomycin sulfate | Yano et al | 1994 | 5/7 (71%) | None |
| | Liu et al | 1996 | 9/11 (82%) | Fever, arrhythmia |
| Mitomycin C | Lee et al | 1994 | 14/20 (70%) | None |
| Cisplatin | Fiorentino et al | 1988 | 3/6 (50%) | Nausea |
| | Tomkowski et al | 1994 | 9/9 (100%) | Nausea, arrhythmia |
| | Tomkowski et al | 1997 | 15/16 (93%) | Nausea, arrhythmia |
| Thiotepa | Colleoni et al | 1998 | 19/23 (83%) | Thrombocytopenia, leukopenia |
| $^{32}$P-colloid | Dempke et al | 1999 | 34/36 (95%) | Tachycardia |

[a] All reports are cited in the References; [b] No. of Pt. showing effectiveness/No. of total Pt; [c] patients consist of lung cancer and other malignant diseases.

### DISCUSSION

The basic standard therapeutic method for relieving and controlling malignant pericardial effusion that accompanies NSCLC is a percutaneous pericardiocentesis followed by drainage through an in-place catheter (Shinkai et al, 1982; Roth et al, 1989). For malignant pericarditis coexisting with various kinds of malignancies, including but not limited to lung cancer, an additional topical instillation of sclerosing or cytotoxic agents is reportedly effective (see references in Table 3). Any retrospective study, however, has failed to demonstrate how such sclerosing agents and cytotoxic agents are superior to standard pericardiocentesis and drainage (Okamoto et al, 1993). This means that a standard treatment for malignant pericardial effusion in patients with NSCLC has not been clearly established until now.

In a first attempt to address this issue, the effectiveness and reliability of percutaneous pericardiocentesis combined with the draining of effused pericardial fluid and topically administering carboplatin were prospectively evaluated in this study. Carboplatin was chosen for this purpose because it is one of the most effective chemotherapeutic agents for NSCLC, as well as cisplatin, in a variety of clinical situations (Lokich and Anderson, 1988; Go and Adjei 1999). Only a few case reports have shown the successful use of the intrapericardial administration of carboplatin for controlling malignant pericarditis (Gotoh et al, 1991; Takahashi et al, 1994). Our present results with carboplatin were satisfactory in terms of both its potency and safety. Eight and one of the ten patients showed a major and a moderate response, respectively, resulting in a 90% success rate according to the previously mentioned criteria. No trace of toxicity was observed in any of the ten patients. With respect of effectiveness, these results with carboplatin are comparable to the above-mentioned studies (Table 3) utilizing tetracycline, doxycycline, bleomycin or cisplatin. In fact, as is the case with bleomycin, the use of carboplatin is highly preferable when considering the harmful side effects typically associated with tetracycline, doxycycline and cisplatin.

The pharmacokinetics data for pericardial fluid and circulating blood clearly appear to support these clinical outcomes. That is, as much as 600 μg/ml of free platinum was retained for as long as 40 minutes during which the catheter was occluded. More impressively, more than 60 μg/ml of that amount was still retained one hour after reopening the catheter (Fig. 1A). Such high concentrations of free platinum existing for 1.5 hours are theoretically enough to kill all cancer cells when considering another in vitro study (Takashashi et al, 1987).

The pharmacokinetics data obtained in a phase I study (van Echo et al, 1984) were comparable to both the plasma $C_{max}$ and AUC presented here. In patients with normal renal function, the plasma $C_{max}$ of this method corresponded to the $C_{max}$ of 5.98 μg/ml for which 77 mg/m$^2$ of carboplatin had been intravenously injected. Similarly, the plasma AUC of this method was comparable to the AUC of 22.6 μg × h/ml for which 77 mg/m$^2$ of carboplatin had been intravenously injected (Table 2).

Such low doses of carboplatin caused few systemic adverse effects in phase I studies (Calvert et al, 1982; Curt et al, 1983) and, to date, there have been no systemic complications in our procedure. Another group (Lerner-Tung et al, 1997) administered 5-fluorouracil followed by cisplatin into the pericardial space of a patient with metastatic breast adenocarcinoma, and the pharmacokinetics of this drug in the pericardial space and circulating plasma were investigated. The results revealed a high concentration within the pericardial sac, with a greatly increased half-life over that of systemic delivery, with little systemic toxicity, resembling our results. Systemically delivering chemotherapeutic agents may be advantageous for controlling disseminated lesions only under particular conditions. However, patients suffering from NSCLC and malignant pericarditis are most likely too advanced in terms of disease. As is commonly the case, systemic delivery of these agents is no longer effective for enhancing the quality of life or for prolonging the survival time of such patients. Therefore, the pharmacokinetics data presented here validates the suitability of our method when applied to the clinical situation of our 10 patients.

To treat patients with malignant pericardial effusion and NSCLC, the present study can not establish whether topically administrating carboplatin followed by drainage is superior to minimal pericardiocentesis followed by drainage, because no prospective comparative study has been conducted. Discussions on the clinical relevance of intrapericardial administration of other sclerosing and cytotoxic agents have also been equivocal. A conclusion could be plainly drawn from directly comparing these

*© 2000 Cancer Research Campaign*

methods to the standard approach. Phase III studies are warranted to compare minimal pericardiocentesis followed by drainage to intrapericardial administration of a sclerosing or cytotoxic agent combined with the minimal approach for controlling malignant pericardial effusion with NSCLC. Carboplatin is one of the most promising candidates to be included in such studies.

### ACKNOWLEDGEMENTS

We wish to thank Drs Akira Suda, Shigenari Ohmori, Yuji Ikeda, Masayuki Ohtaki, Atsuko Tokuda, Hiroshi Miyazawa, and Takaaki Sugimoto, Department of Chest Medicine, Chiba University School of Medicine, for their assistance. The study was financially supported by grants from the Ministry of Education of Japan.

### REFERENCES

Calvert AH, Harland SJ, Newell DR, Siddik ZH, Jones AC, McElwain TJ, Raju S, Wiltshaw E, Smith IE, Baker JM, Peckham MJ and Harrap KR (1982) Early clinical studies with cis-diammine-1,1-cyclobutane dicarboxylase platinum II. *Cancer Chemother and Pharmacol* **9**: 140–147

Celermajor DS, Boyer M, Bailey BP and Tattersall MHN (1991) Pericardiocentesis for symptomatic malignant pleural effusions: a study of 36 patients. *Med J Aust* **154**: 19–22

Colleoni M, Martinelli G, Beretta F, Marone C, Gallino A, Fontana M, Graffeo R, Zampino G, De Pas T, Cipolla G, Martinoni C and Goldhirsch A (1998) Intracavitary chemotherapy with thiopeta in malignant pericardial effusions: An active and well-tolerated regimen. *JCO* **16**: 2371–2376

Curt GA, Grygiel JJ, Corden BJ, Ozols RF, Weiss RB, Tell DT, Myers CE and Collins JM (1983) A phase I and pharmacokinetic study of diammine-cyclobutanedicarboxylatoplatinum (NSC 241240). *Cancer Res* **43**: 4470–4473

Davis S, Rambotti P and Grignani F (1984) Intrapericardial tetracycline sclerosis in the treatment of malignant pericardial effusion: an analysis of thirty-three cases. *J Clin Oncol* **2**: 631–636

Dempke W and Firusian N (1999) Treatment of malignant pericardial effusion with $^{32}$P-colloid. *Br J Cancer* **80**: 1955–1957

Fiorentino MV, Daniele O, Morandi P, Aversa SML, Ghiotto C, Paccgnella A and Fornasiero A (1988) Intrapericardial instillation of platin in malignant pericardial effusion. *Cancer* **62**: 1904–1906

Go RS and Adjei AA (1999) Review of the comparative pharmacology and clinical activity of cisplatin and carboplatin. *JCO* **17**: 409–422

Gotoh T, Fujita Y, Uchida H, Arimoto T, Sakai M, Kida T, Fujii T, Hiramori N, Iwasaki Y, Nakamura T and Nakagawa M (1991) Successful treatment with intrapericardial administration of carboplatin in a case of squamous cell lung cancer with malignant pericardial effusion. *Jpn J Cancer Chemother* **18**: 2337–2340

Herrstedt J, Clementsen P and Hansen P (1992) Increased myelosuppression during cytostatic treatment and pleural effusion in patients with small cell lung cancer. *Eur J Cancer* **28A**: 1070–1073

Kitamura S, Wagai F, Izumi T, Sugiyama Y and Kosaka K (1981) Treatment of carcinomatous pericarditis with doxycycline: intrapericardial doxycycline for control of malignant pericardial effusion. *Curr Therapeut Res* **30**: 589–596

Lee LN, Yang PC, Chang DB, Yu CJ, Ko JC, Liaw YS, Wu RG and Luh KT (1994) Ultrasound guided pericardial drainage and intrapericardial instillation of mitomycin C for malignant pericardial effusion. *Thorax* **49**: 594–595

Lerner-Tung MB, Chang AYC, Ong LS and Kreiser D (1997) Pharmacokinetics of intrapericardial administration of 5-fluorouracil. *Cancer Chemother Pharmacol* **40**: 318–320

Liu G, Crump M, Gross PE, Dancey J and Shepherd FA (1996) Prospective comparison of the sclerosing agents doxycycline and bleomycin for the primary management of malignant pericardial effusion and cardiac tamponade. *J Clin Oncol* **14**: 3141–3147

Livingston RB, Joseph D, McCracken JD, Trauth CJ and Chen T (1982) Isolated pleural effusion in small cell lung carcinoma: favorable prognosis. *Chest* **81**: 208–211

Lokich J and Anderson N (1998) Carboplatin versus cisplatin in solid tumors: An analysis of the literature. *Ann Oncol* **9**: 13–21

Maher EA, Shepherd FA and Todd TJR (1996) Pericardial sclerosis as the primary management of malignant pericardial effusion and cardiac tamponade. *J Thorac Cardiovasc Surg* **112**: 637–643

Okamoto H, Shinkai T, Yamakido M and Saijo N (1993) Cardiac tamponade caused by primary lung cancer and the management of pericardial effusion. *Cancer* **71**: 93–98

Patton TF, Himmelstein KJ, Belt R, Bannister SJ, Sternson LA and Repta AJ (1978) Plasma levels and urinary excretion of filterable platinum species following bolus injection and iv infusion of cis-dichlorodiammineplatinum (II) in man. *Cancer Treat Rep* **62**: 1359–1362

Pera MF Jr and Harde HCr (1977) Analysis for platinum in biological material by flameless atomic absorption spectrometry. *Clin Chem* **23**: 1245–1249

Press OW and Livingston R (1987) Management of malignant pericardial effusion and tamponade. *JAMA* **257**: 1088–1092

Roth JA, Ruckdeschel JC and Weisenburger TH (1989) Comparison of therapy of malignant tumors involving the pericardium. *Thoracic Oncol* **34**: 504–512

Shepherd FA, Morgan C, Evans WK, Ginsberg JF, Watt D and Murphy K (1987) Medical management of malignant pericardial effusion by tetracycline sclerosis. *Am J Cardiol* **60**: 1161–1166

Shinkai T, Tominaga K, Saijo N, Eguchi K, Shimizu E, Shibuya M, Shimabukuro Z, Saito Y, Tsuchiya R and Niitani H (1982) The incidence of cardiac metastasis in primary lung cancer and the management of malignant pericardial effusion. *Jpn J Clin Oncol* **12**: 23–32

Smith FE, Lane M and Hudgins PT (1974) Conservative management of malignant pericardial effusion. *Cancer* **33**: 47–57

Souquet PJ, Chauvin F, Boissel JP, Cellerio R, Cormier Y, Ganz PA, Kaasa S, Pater JL, Quoix E, Rapp E, Tumarello D, Williams J, Woods BL and Bernard JP (1993) Polychemotherapy in advanced non small lung cancer: a meta-analysis. *Lancet* **342**: 19–21

Takahashi H, Sasaki Y, Saijo N, Sakurai M, Nakao H, Nakagawa K, Hoshi A, Jett JR and Hong WS (1987) In vitro colony inhibition of carboplatin against stomach and lung cancer cell lines in comparison with cisplatin. *Cancer Chemother and Pharmacol* **19**: 197–200

Takahashi J, Kinomura S, Abe Y, Yoshioka S, Yambe T, Ono S, Ito H, Fukuda H, Yamada K and Sato T (1994) Two cases of malignant effusion treated successfully by loco-regional administration of carboplatin. *Jpn J Cancer Chemother* **21**: 2817–2820

Tomkowski W, Szturmowicz M, Fijalkowska A, Filipecki S and Chojak EF (1994) Intrapericardial cisplatin for the management of patients with large malignant pericardial effusion. *J Cancer Res Clin Oncol* **120**: 434–436

Tomkowski WZ and Filipecki S (1997) Intrapericardial cisplatin for the management of patients with large pericardial effusion in the course of the lung cancer. *Lung Cancer* **16**: 215–222

Vaitkus PT, Hermann HC and LeWinter MM (1994) Treatment of malignant pericardial effusions. *JAMA* **272**: 59–64

van Echo DA, Egorin MJ, Whitacre MY, Olman EA and Aisner J (1984) Phase I clinical and pharmacologic trial of carboplatin for 5 days. *Cancer Treat Rep* **68**: 1103–1114

Woods RL, Williams CJ, Levi J, Page J, Bell D, Byrne M and Karestes ZL (1990) A randomized trial of cisplatin and vindesine versus supportive care only in advanced non-small cell lung cancer. *Br J Cancer* **61**: 608–611

Yano T, Yokoyama H, Inoue T, Takanashi N, Asoh H and Ichinose Y (1994) A simple technique to manage malignant pericardial effusion with a local instillation of bleomycin in non-small cell carcinoma of lung. *Oncology* **51**: 507–509

 *© 2000 Cancer Research Campaign*
